# Infliximab Rescue in Acute Severe Ulcerative Colitis Complicated by Clostridium Difficile Infection: A Case Series

**DOI:** 10.7759/cureus.19019

**Published:** 2021-10-25

**Authors:** Srdjan Markovic, Marijana Jankovic, Ana Kalaba, Branimir Zogovic, Slobodan S Sreckovic

**Affiliations:** 1 Gastroenterology and Hepatology, University Hospital Medical Center "Zvezdara", Belgrade, SRB; 2 Department of General Surgery, Royal Prince Alfred Hospital, New South Wales, AUS

**Keywords:** metronidazole, fidaxomixin, vancomycin, infliximab, acute severe ulcerative colitis, clostridium difficile infection, anti‑tnf

## Abstract

Relapses in inflammatory bowel disease (IBD) complicated by *Clostridium difficile* infection (CDI) are associated with significant morbidity and mortality. CDI can exacerbate the course of IBD and may result in prolonged hospitalizations, admissions to intensive care, surgical interventions, or even death. Early detection and aggressive treatment with systemic corticosteroids or biologics such as infliximab are often needed in severe presentations.

Five cases of relapsed ulcerative colitis complicated by fulminant CDI were retrospectively reviewed. Biological therapy with infliximab was initiated upon multidisciplinary team assessment as the cases were resistant to standard IBD therapy.

All five patients improved clinically and avoided early surgical intervention. Some required prolonged therapy with infliximab to achieve remission.

Early recognition of CDI and aggressive therapy with biologics may be required to avoid complications in the IBD patients experiencing a relapse. Infliximab therapy should be considered early on, especially where the disease is resistant to standard therapy.

## Introduction

*Clostridium difficile* infection (CDI) is a recognized factor of relapse in inflammatory bowel disease (IBD) [1-3]. While the exact mechanism leading to CDI is unknown, medications and mucosal micro-environmental changes are implicated in the pathogenesis. Antibiotics (e.g., fluoroquinolones, clindamycin, cephalosporins, and penicillin), proton pump inhibitors, corticosteroids, and immunosuppressors are the most common medications associated with CDI in IBD [4]. Immunomodulators, which play an important role in the control of the disease course in IBD, have not been associated with CDI [4]. In the theory of micro‑environmental changes, it is suggested that epithelial changes provide opportunistic conditions for superseding bacterial infections [5].

Differentiating between CDI and IBD relapse is challenging as symptoms overlap. CDI in IBD patients can have a fulminant course; therefore, early detection and management are essential in order to minimize complications, especially in asymptomatic carriers and immunosuppressed patients [6]. IBD patients with protracted clinical course, recent surgery, and sepsis are especially vulnerable and may experience unfavorable outcomes [5,7]. Any IBD patient presenting with diarrhea or colitis‑like picture should be tested for *Clostridium difficile* toxin in the stool [3,8]. The highest diagnostic accuracy can be achieved using a combination of enzyme immunoassay (EIA) with cell cytotoxin assay for glutamate dehydrogenase (GDH) [9,10].

Oral vancomycin is the first line of treatment for CDI eradication, given its efficacy of 97%. Therapy should be supplemented with oral or intravenous metronidazole [3,11]. Both metronidazole and vancomycin have good luminal bioavailability in the colon and rectum. Fidaxomicin is a narrow-spectrum antibiotic that was shown to achieve a long-lasting remission in initial and mild and moderate CDI cases. Fidaxomicin is a safe and effective alternative to vancomycin. Both vancomycin and fidaxomicin should be used in severe CDI as metronidazole is no longer recommended [12-14].

In this work, we present the management of five patients with ulcerative colitis that relapsed as a result of fulminant CDI.

## Case presentation

We retrospectively reviewed five cases of patients with ulcerative colitis whose relapse was complicated by fulminant CDI (Table 1). Three male and two female patients were diagnosed with fulminant ulcerative colitis using the Truelove-Witts clinical severity score for ulcerative colitis [15]. The average patient age was 38 years, while the average duration of illness was 47 months. All patients had a similar disease course and were subjected to standardized diagnostic and therapeutic protocols. *Clostridium difficile* was detected with standard laboratory tests. The patients were treated at the Department of Gastroenterology, Clinical Center Zvezdara (Belgrade, Serbia) from September 2017 to September 2018. Our center oversees and manages around 1500 IBD patients. Four hundred patients in this cohort are treated with biologics such as anti-TNFα antibodies. Here, we present the most severe cases of IBD relapse complicated by CDI that required hospitalization. The case series was completed in accordance with the Declaration of Helsinki.

**Table 1:** Clinical presentation, demographics, treatment, and outcome of patients with ulcerative colitis and Clostridium difficile infection UC, Ulcerative colitis; BT, body temperature; HR, heart rate; Hb, hemoglobin; CRP, C-reactive protein; AZA, azathioprine; PPI, proton pump inhibitors.

Patient	Demographics	Montreal Classification	Truelove-Witts Severity Index on Admission	Mayo Endoscopic Subscore on Admission	Therapy	Treatment Outcome
Case #1	Female, 28 years old	E3, extensive UC	Severe; bowel movements per day: 10/24 h; blood in stool: visible blood, BT 37.5°C; HR 100/min; Hb 10.8 g/dl, CRP 81 mg/l	3 - Severe	Dexamethasone; mesalamine oral and topical, AZA, metronidazole, vancomycin, teicoplanin, probiotic, PPI, and infliximab	Complete remission with mucosal healing
Case #2	Male, 42 years old	E3, extensive UC	Severe; bowel movements per day: 7/24 h; blood in stool: visible blood; BT 36.4°C; HR 75/min; Hb 12.5 g/dl; CRP 88 mg/l	3 - Severe	Dexamethasone, mesalamine oral and topical; AZA; metronidazole; vancomycin, probiotic, PPI, and infliximab	Complete remission and histological remission
Case #3	Male, 40 years old	E3, extensive UC	Severe; bowel movements per day: 12/24 h; blood in stool: visible blood; BT 36.9°C; HR 110/min; Hb 13.0 g/dl; CRP 185 mg/l	3 - Severe	Dexamethasone, mesalamine oral and topical; AZA, metronidazole, vancomycin, probiotic, PPI, and infliximab	Complete remission with mucosal healing
Case #4	Male, 46 years old	E3, extensive UC	Severe; bowel movements per day: 10/24 h; blood in stool: visible blood; BT 39.0°C; HR 95/min; Hb 11.0 g/dl; CRP 165 mg/l	3 - Severe	Dexamethasone, mesalamine oral and topical; AZA; metronidazole, vancomycin, probiotic, PPI, and infliximab	Complete remission with mucosal healing
Case #5	Female, 38 years old	E3, extensive UC	Severe; bowel movements per day: 20/24 h; blood in stool: visible blood; BT 36.8°C; HR 84/min; Hb 7.9 g/dl; CRP 152 mg/l	3 - Severe	Dexamethasone; mesalamine oral and topical; AZA; metronidazole, vancomycin, probiotic, PPI, and infliximab	Complete remission with histological remission

Case 1: A 28-year-old woman with a three-year history of mild and stable ulcerative colitis maintained with mesalamine

The patient presented with 10 days of bloody diarrhea, abdominal pain, and fever. She underwent colonoscopy that showed moderate ulcerative colitis (Mayo subscore of 2) [16]. The patient was discharged on a two-week course of oral mesalamine 4.8 g daily, mesalamine enema, and 40 mg prednisolone. At the end of the 14-day course, she presented to our hospital with active disease. Vital signs revealed borderline tachycardia of 100 beats per min (bpm). Clinical examination demonstrated abdominal distension. Blood workup showed elevated white blood cell count (WBC = 17 × 10^9^/L), C-reactive protein (CRP = 81), and platelet count (PLT = 582 × 10^9^/L), while the hemoglobin (13.1 g/dL) and albumin (32 g/L) were normal. Cytomegalovirus (CMV) IgM, Epstein-Barr virus (EBV) IgM, anti-hepatitis C virus (anti-HCV) Ab, anti-HIV Ab, hepatitis B surface antigen (HbsAg), and QuantiFERON tests were negative, while glutamate dehydrogenase (GDH) test for *Clostridium difficile* returned positive on day three of admission. Erect abdominal x-rays showed non-specific gaseous distention of the small bowel. Repeat colonoscopy demonstrated severe ulceration from the rectosigmoid junction, extending beyond the limit of endoscopy (Mayo subscore of 3). The patient was admitted and commenced on intravenous (i.v.) steroids, vancomycin, and metronidazole. Despite the treatment, the patient continued to have frequent stools and low-grade fevers. Oral teicoplanin 200 mg daily was added to the treatment regimen on the advice of the infectious diseases team. Surgical clearance was obtained as therapy with infliximab was considered. Once PCR for *Clostridium difficile* and *Clostridium difficile* Toxin A and B returned negative, infliximab was commenced, and the patient had an excellent therapeutic response after the first dose. Therapy with infliximab and mesalazine with regular monitoring is ongoing, while azathioprine was discontinued after two years due to drug intolerance. The patient achieved complete remission with mucosal healing during the last follow-up in September 2021.

Case 2: A 42-year-old male with a recent diagnosis of moderate ulcerative colitis (Mayo subscore of 2)

The patient was admitted to the hospital with 20 days of bloody diarrhea, abdominal pain, and fever. On examination, the patient had notable abdominal distension. Laboratory investigations showed raised WBC count at 22 × 10^9^/L and CRP at 88 mg/L. The erect abdominal x-ray was unremarkable. Colonoscopy demonstrated severe left colonic ulceration (Mayo subscore of 3). CMV IgM, EBV IgM, Anti-HCV Ab, anti-HIV Ab, HbsAg, and QuantiFERON tests were negative, while EBV IgG and GDH test for *Clostridium difficile* were positive. Upon admission, the patient was started on i.v. steroids and azathioprine (2.5 mg/kg), vancomycin, and metronidazole. The infectious diseases team was consulted as the patient continued to have frequent stools despite the therapy. Once PCR for *Clostridium difficile* and *Clostridium difficile *Toxin A and B returned negative, infliximab was commenced, and the patient had an excellent therapeutic response after the first dose. After 14 weeks of therapy, the patient achieved clinical and laboratory remission at which point the infliximab trough level was 8.6 microg/mL, and there were no antibodies detected. After the third induction dose of infliximab, maintenance therapy with infliximab, oral mesalamine, and azathioprine was continued for 18 months. One year within the maintenance protocol, the patient achieved complete clinical and histological remission.

Case 3: A 40-year-old male with long-standing ulcerative colitis maintained with mesalazine and azathioprine

The patient had a relapse of severe ulcerative colitis in January 2018 (Mayo subscore of 3) and was treated with i.v. corticosteroids and azathioprine (2.5 mg/kg). He relapsed again in June 2018 when he presented with seven days of bloody diarrhea (around 15 stools a day), abdominal pain, and lethargy. On presentation, he was tachycardic (110 bpm) and had non‑peritonitic abdominal tenderness on examination. Workup showed significantly elevated CRP at 185 mg/L and mildly raised WBC count of 12.8 × 10^9^/L. The abdominal x-ray was unremarkable. Flexible sigmoidoscopy demonstrated severe ulceration from the rectosigmoid junction extending beyond the limit of endoscopy (Mayo subscore of 3). The patient was commenced on i.v. steroids and azathioprine (2.5 mg/kg daily). CMV IgM, EBV IgM, anti-HCV Ab, anti-HIV Ab, HbsAg, and QuantiFERON tests were negative. As *Clostridium difficile* test returned positive on day five of hospitalization, vancomycin and metronidazole were added to the treatment protocol. The infectious diseases team was consulted as the patient continued to have frequent stools despite the therapy. Once PCR for *Clostridium difficile* and *Clostridium difficile* Toxin A and B returned negative, infliximab was started, and the patient experienced an improvement in both the clinical picture and laboratory findings. After 14 weeks of infliximab therapy, the patient achieved clinical remission with infliximab trough level > 12 microg/mL and absence of antibodies. Mucosal healing was achieved with maintenance therapy that included infliximab, azathioprine, and oral mesalamine. Complete remission was noted during the last follow-up in August 2021.

Case 4: A 46-year-old male with a recent diagnosis of ulcerative colitis controlled with mesalazine

This patient presented to our outpatient clinic in August 2018 with mild diarrhea. This was his first relapse since the diagnosis was made in December 2017. *Clostridium difficile* was isolated from a stool sample, and vancomycin was commenced. The patient represented again in September with abdominal pain, high-grade fever, and up to 10 loose bloody motions a day. Vital signs showed borderline tachycardia at 100 bpm. On examination, the patient had abdominal distention and non-peritonitic tenderness. Blood tests showed elevated WBC count, CRP, and platelets at 15.6 × 10^9^/L, 165 mg/L, and 550 × 10^9^/L, respectively. The erect chest x‑ray did not show pneumoperitoneum. Flexible sigmoidoscopy demonstrated severe ulceration from the rectosigmoid junction, extending beyond the limit of endoscopy (Mayo subscore of 3). CMV IgM, EBV IgM, anti-HCV Ab, anti-HIV Ab, HbsAg, and QuantiFERON tests were negative. GDH test for *Clostridium difficile* was positive on the first day of hospitalization. The patient was started on 4 mg i.v. dexamethasone TDS, oral and rectal mesalazine, azathioprine (2.5 mg/kg daily), vancomycin, and metronidazole. The infectious diseases team was consulted as the patient continued to have loose stools despite the therapy. Once PCR for *Clostridium difficile* and *Clostridium difficile* Toxin A and B returned negative, infliximab was started, and clinical remission was achieved. Maintenance therapy with infliximab, oral mesalazine, and azathioprine was continued for three years. The patient was in complete clinical, laboratory, and endoscopic remission during the last follow-up in September 2021. Azathioprine was discontinued from the maintenance therapy after three years due to a pathological hepatogram.

Case 5: A 38-year-old female with a short history of ulcerative colitis successfully controlled with a brief course of sulfasalazine

The patient presented to our clinic with moderate bloody diarrhea, abdominal pain, and fever. The patient was deemed safe for outpatient treatment; however, she represented three days later with symptoms of severe ulcerative colitis. On examination, abdominal distension and non-peritonitic tenderness were noted. Blood tests showed significant anemia (7.9 g/dL) and elevated CRP (152 mg/L). Erect abdominal x-rays showed non-specific gaseous distention. Flexible sigmoidoscopy demonstrated severe ulceration from the rectosigmoid junction extending beyond the limit of endoscopy (Mayo subscore of 3). CMV IgM, EBV IgM, anti-HCV Ab, anti-HIV Ab, HbsAg, and QuantiFERON tests were negative. GDH test *Clostridium difficile* was positive. The patient was started on i.v. steroids, vancomycin, and metronidazole. The infectious diseases team was consulted as the patient continued to experience frequent stools despite the therapy. Once PCR for *Clostridium difficile* and *Clostridium difficile* Toxin A and B returned negative, infliximab was started, and the patient had an excellent therapeutic response after the first dose. After 14 weeks, the patient had clinical remission and optimal trough level without the presence of antibodies. Maintenance therapy with infliximab, oral mesalazine, and azathioprine was continued for three years. Azathioprine was excluded after two years as mucosal healing was achieved. The maintenance therapy with infliximab and mesalazine was discontinued in November 2020 as the patient was in stable clinical and histological remission for two years.

## Discussion

In this retrospective case series, we found that early infliximab treatment in severe CDI cases may yield a turning point in relapsed IBD patients. The incidence of CDI complicating IBD relapses is low; however, the associated morbidity and mortality are high. A large study of 124,570 hospitalizations that looked at the incidence of CDI, IBD, or both CDI in IBD found that only 2.3% of inpatients were diagnosed with both CDI and IBD. However, patients with both CDI and IBD had extended admissions and four times greater mortality than patients with IBD (OR 4.7) or *Clostridium difficile* alone (OR 2.2). Among IBD patients, patients with ulcerative colitis had higher complication rates requiring emergency surgery when compared to Crohn’s disease patients [1]. The majority of IBD patients with a prior CDI are more likely to require early initiation of biologics in order to avoid significant complications. In a cohort study of 654 patients, 57% of IBD patients with CDI required early biologics in contrast to 37% of IBD patients without CDI [17] to prevent severe aggressive IBD. For these reasons, our patients with severe IBD and concurrent CDI were started on infliximab. In terms of IBD severity and CID, similar trends were noted in Canada and other European countries [1].

Early suspicion and microbiological confirmation of CDI are essential. Enzyme immunoassay (EIA) testing for CD toxin A and B is the diagnostic test of choice, given its availability, usability, rapidity, and affordability. In contrast to the cell-culture cytotoxin assay, which is the gold standard test for CDI detection, EIA has lower sensitivity (63%-94%) and specificity (75%-100%) [2]. Therefore, a combination of EIA with cell-culture cytotoxin assay targeting glutamate dehydrogenase (GDH) is recommended to achieve the highest diagnostic accuracy. EIA for GDH is a rapid screening tool with a high negative predictive value, while the cell cytotoxin assay confirms GDH-positive stool samples [9,10].

Once CDI is suspected, early eradication with vancomycin should be commenced [3,11]. Vancomycin has succeeded metronidazole as the first choice in the treatment of CDI as CDI is becoming more refractory to metronidazole treatment [18]. Recent studies suggest that metronidazole failures range between 22% and 26%, compared to only 10% in the 1990s [18]. Fidaxomicin is a potent narrow-spectrum antibiotic introduced in 2011 that can be used in initial presentations and first CDI recurrences [19]. Fidaxomicin and vancomycin are superior to metronidazole for CDI and are now recommended for severe CDI [12-14]. Fidaxomicin may not be readily available due to the high price cost [19].

IBD patients with concurrent CDI are more likely to deteriorate clinically as a result of resistance to standard treatment protocols and often require intensive care admissions [1,2,8]. Actively looking for severe CDI (defined by leukocytosis, acute kidney injury, or low albuminemia) and starting treatment early and aggressively minimize adverse outcomes [2,20]. Therefore, it is sensible to consider CDI as an IBD severity marker in the treatment algorithm.

In clinical practice, CDI in IBD patients is treated with both antibiotics and immunosuppression/immunomodulators. There are no guidelines to date, and the literature is conflicting [5]. For example, a study of 155 patients compared the effectiveness of combined immunomodulators (prednisone, thiopurines, methotrexate, cyclosporine, tacrolimus) and antibiotics versus antibiotics alone in IBD patients with CDI. The effectiveness was compared against predefined primary outcomes: megacolon, colonic perforation, cardiogenic shock requiring vasopressors, intubation and ventilation, mortality, or colectomy within three months of admission. All primary outcomes occurred in the antibiotics and immunomodulators treatment group. This was 8% of all patients (12 of 104) or 12% (12 of 155) of the patients in the combined group. Moreover, the use of more than one immunomodulator further increased the risk of side effects. Interestingly, no adverse outcomes were noted in the patients treated with antibiotics only [4]. In contrast, a retrospective study assessing IBD patients with CDI showed that immunomodulators, corticosteroids, or antitumor necrosis factor agents did not predict adverse outcomes. More reliable indicators for mortality and colectomy were low albumin, anemia, and acute renal failure [20].

In the absence of prospective data, using antibiotics as the only treatment regimen in managing CDI in severe IBD relapse while withholding immunosuppression cannot be recommended. In the unwell patient, it is reasonable to start corticosteroids and even escalate to immunosuppressive therapy once it is established that symptoms cannot be controlled with vancomycin, metronidazole, and/or fidaxomicin. Therefore, immunosuppressive therapy should commence, and the patients should be monitored closely for subtle signs of deterioration [5].

## Conclusions

CDI in IBD patients may be used as an indicator for a complicated disease course. Clinicians must have a low threshold in suspecting and diagnosing CDI in IBD relapses in order to avert a complicated or fatal clinical course. If the relapse is not controlled with antibiotics, escalation to immunosuppressive and biological therapy may be appropriate. Patients on immunosuppressive and biological should be monitored closely for deterioration.

## References

[1] Ananthakrishnan AN, McGinley EL, Binion DG (2008). Excess hospitalisation burden associated with Clostridium difficile in patients with inflammatory bowel disease. Gut.

[2] Cohen SH, Gerding DN, Johnson S (2010). Clinical practice guidelines for Clostridium difficile infection in adults: 2010 update by the society for healthcare epidemiology of America (SHEA) and the infectious diseases society of America (IDSA). Infect Control Hosp Epidemiol.

[3] Surawicz CM, Brandt LJ, Binion DG (2013). Guidelines for diagnosis, treatment, and prevention of Clostridium difficile infections. Am J Gastroenterol.

[4] Ben-Horin S, Margalit M, Bossuyt P (2009). Combination immunomodulator and antibiotic treatment in patients with inflammatory bowel disease and clostridium difficile infection. Clin Gastroenterol Hepatol.

[5] Khanna S, Shin A, Kelly CP (2017). Management of Clostridium difficile Infection in inflammatory bowel disease: expert review from the clinical practice updates committee of the AGA institute. Clin Gastroenterol Hepatol.

[6] Sinh P, Barrett TA, Yun L (2011). Clostridium difficile infection and inflammatory bowel disease: a review. Gastroenterol Res Pract.

[7] Butala P, Divino CM (2010). Surgical aspects of fulminant Clostridium difficile colitis. Am J Surg.

[8] Debast SB, Bauer MP, Kuijper EJ (2014). European society of clinical microbiology and infectious diseases: update of the treatment guidance document for Clostridium difficile infection. Clin Microbiol Infect.

[9] Reller ME, Lema CA, Perl TM, Cai M, Ross TL, Speck KA, Carroll KC (2007). Yield of stool culture with isolate toxin testing versus a two-step algorithm including stool toxin testing for detection of toxigenic Clostridium difficile. J Clin Microbiol.

[10] Ticehurst JR, Aird DZ, Dam LM, Borek AP, Hargrove JT, Carroll KC (2006). Effective detection of toxigenic Clostridium difficile by a two-step algorithm including tests for antigen and cytotoxin. J Clin Microbiol.

[11] McDonald LC, Gerding DN, Johnson S (2018). Clinical practice guidelines for clostridium difficile infection in adults and children: 2017 update by the infectious diseases society of America (IDSA) and society for healthcare epidemiology of America (SHEA). Clin Infect Dis.

[12] Zar FA, Bakkanagari SR, Moorthi KM, Davis MB (2007). A comparison of vancomycin and metronidazole for the treatment of Clostridium difficile-associated diarrhea, stratified by disease severity. Clin Infect Dis.

[13] Stevens VW, Nelson RE, Schwab-Daugherty EM (2017). Comparative effectiveness of vancomycin and metronidazole for the prevention of recurrence and death in patients with Clostridium difficile infection. JAMA Intern Med.

[14] Gentry CA, Nguyen PK, Thind S, Kurdgelashvili G, Skrepnek GH, Williams RJ 2nd (2019). Fidaxomicin versus oral vancomycin for severe Clostridium difficile infection: a retrospective cohort study. Clin Microbiol Infect.

[15] TR SC, WI LJ (1955). Cortisone in ulcerative colitis; final report on a therapeutic trial. Br Med J.

[16] Travis SP, Schnell D, Krzeski P (2013). Reliability and initial validation of the ulcerative colitis endoscopic index of severity. Gastroenterology.

[17] Gillespie W, Marya N, Fahed J, Leslie G, Patel K, Cave DR (2017). Clostridium difficile in inflammatory bowel disease: a retrospective study. Gastroenterol Res Pract.

[18] Pepin J, Alary ME, Valiquette L (2005). Increasing risk of relapse after treatment of Clostridium difficile colitis in Quebec, Canada. Clin Infect Dis.

[19] Bartsch SM, Umscheid CA, Fishman N, Lee BY (2013). Is fidaxomicin worth the cost? An economic analysis. Clin Infect Dis.

[20] Ananthakrishnan AN, Guzman-Perez R, Gainer V (2012). Predictors of severe outcomes associated with Clostridium difficile infection in patients with inflammatory bowel disease. Aliment Pharmacol Ther.

